# Retraction: Quercetin inhibits tumorigenesis of colorectal cancer through downregulation of hsa_circ_0006990

**DOI:** 10.3389/fphar.2023.1252183

**Published:** 2023-07-06

**Authors:** Frontiers Editorial Office

Following publication, the authors contacted the Editorial Office to request the retraction of the cited article, stating that cell lines used in the article were contaminated by *Mycoplasma*. It was confirmed by the authors when they tried to repeat their experiments unsuccessfully. The findings and conclusions reported in the article are no longer supported by the data. An investigation was conducted in accordance with Frontiers’ policies that confirmed this; therefore, the article has been retracted.

This retraction was approved by the Chief Editors of Frontiers in Pharmacology and the Chief Executive Editor of Frontiers. The authors agree to this retraction.

